# Recent Advances in Fermented Milk and Meat Products—Quality, Nutritional Value and Safety

**DOI:** 10.3390/ani13132048

**Published:** 2023-06-21

**Authors:** Anna Maria Ogrodowczyk, Monika Modzelewska-Kapituła

**Affiliations:** 1 Department of Immunology and Food Microbiology, Institute of Animal Reproduction and Food Research of Polish Academy of Sciences, Tuwima 10, 10-748 Olsztyn, Poland; 2 Department of Meat Technology and Chemistry, Faculty of Food Sciences, University of Warmia and Mazury in Olsztyn, Plac Cieszyński 1, 10-719 Olsztyn, Poland; monika.modzelewska@uwm.edu.pl

Products of animal origin have always been very popular among consumers due to their nutritional and sensory value. At the same time, their microbiological quality has always caused many technological and safety-associated problems. In recent years, microbiological awareness has been growing and hygiene conditions in farms and processing companies have decreased bacterial biodiversity and quantity in raw animal-derived materials. Still, many factors like animal diet and welfare, milking conditions, slaughter, processing, ageing, and storage influence the microbiological quality of animal-derived raw materials. The adaptation of various animal species to produce milk or meat also presents scientists with new challenges. Easier access to modern identification techniques can provide powerful tools for exploring this issue.

The use of fermentation with selected starters has long been considered an excellent tool to improve the safety and nutritional/functional properties of food by means of the elimination of microbial contamination, the synthesis of bioactive compounds, detoxification, or via the degradation of anti-nutritional compounds while ameliorating their sensory and technological features. Spontaneous fermentation and microbiological changes, especially at the stage of the maturation and ageing of products, is also a phenomenon that require monitoring.

The Special Issue “Recent Advances in Fermented Milk and Meat Products—Quality, Nutritional Value and Safety” aimed to collect the latest research related to the development of new fermented products and to improve the microbiological quality, nutritional value and safety of animal-derived food products.

This Special Issue included eleven research articles that covered various topics, including an analysis of milk’s microbiological quality and safety in the context of its nutrition and applicability for fermented product production [1,2,3,4], the development of novel milk- and meat-based food [5,6], the influence of animal nutrition, material chemical composition and applied processing on the nutritional value of final fermented products [7,8,9], and the use of raw materials of animal origin as a source of potentially probiotic strains [10]. In addition, there are also works examining consumer preferences and sensory analyses of novel products [6,11]. In this Special Issue, goat’s milk turned out to be an attractive raw material in the context of its microbial profile [4,10] but also its chemical composition [8,9]. In addition, this Special Issue also describes the studies of milk from various breeds of cows and other species, including human milk, but also material at various stages of maturity [1,4]. The research was carried out using high-throughput, omics techniques [1,8]. What is more, in vitro but also in silico analyses are used to assess the safety of microbial proteins and strains [1,10].

In the current Special Issue, for the first time, the subject of the microbiological safety of raw animal-derived material was discussed in terms of the allergenicity/immunoreactivity of proteins of microbial origin [1]. In this study, raw cow’s milk samples from Holstein and Jersey breeds, commercially available pasteurized milk, and milk from three human donors in the late lactation phase were subjected to chemical, microbiological, proteomic and molecular analyzes. Microorganisms from the milk material were recovered and cultured, and their immunoreactivity was tested with human sera. Milk and microbial proteins were identified with LCMS and subjected to an in silico analysis of their various activities. It was confirmed that the microbial proteins of human milk show a stronger IgE and IgG immunoreactivity in the tests with human sera compared to the microbial proteins of cow’s milk. There were no significant differences between the composition of microbial proteins in the material obtained from different cattle breeds. The MS-identification and in silico tests of microbial proteins confirmed the presence of GAPDH, GroEL, enolase, ABC transporters and elongation factor Tu with confirmed IgE-immunoreactivity and over 17 proteins with numerous different biological functions including antioxidant, neuroprotective, anti-inflammatory potential. It was concluded that bacterial proteins of culturable, milk-derived bacteria can induce humoral antibodies of E and G class-related reactions. Even though microbial proteins in milk material are not abundant, the evaluation of their immunoreactivity could be implemented as a new food safety criterion.

The subject of food safety also appeared in the works in the context of antimicrobial resistance and sensitivity. The objectives of the Elafify et al.’s [2] study were to determine the antimicrobial resistance and profiling of the virulence genes of the isolated non O_157_:H_7_ *E. coli*, especially Shiga toxin-producing *Escherichia coli* (STEC), in retailed milk and dairy products in Egypt. In this study, the evaluation of the inhibitory effects of D-tryptophan against *E. coli* O_26_:H_11_ was also performed. The results revealed that 20% (30/150) of the samples were STEC-positive, with 64 isolates harboring some virulent genes, such as Stx1, Stx2, eaeA, and hylA. Serological identification revealed four different pathotypes belonging to EPEC, ETEC, EHEC, and EIEC. Antimicrobial susceptibility testing revealed that 100%, 98.44%, 92.19%, 71.87%, 65.63% and 64.06% of the isolates had a resistance to tetracycline, oxacillin, erythromycin, nalidixic acid, sulphamethoxazol, and ampicillin, respectively. D-tryptophan addition (40 mM) to *E. coli* O_26_:H_11_-inoculated soft cheese and ice cream revealed a significant reduction (*p* < 0.05) in bacterial growth.

In the study by Zarzecka et al. [3], the influence of lactic acid bacteria (LAB) carriers of antibiotic resistance genes on the antimicrobial efficacy of high-pressure processing was tested. High-pressure processing is one of the most promising, novel, nonthermal methods of food preservation, and is increasingly being used in the food industry. If products made with starter cultures are subjected to high-pressure treatment, the process parameters should be selected so as not to eliminate all microorganisms in the product. Among 16 strains (*L. lactis* ssp. *lactis*: n = 8; *Latilactobacillus curvatus*: n = 1, *Lacticaseibacillus paracasei*: n = 2, *Lactiplantibacillus paraplantarum*: n = 2, *Lactobacillus delbrueckii*: n = 1, and *Lactobacillus* sp.: n = 2), 8 of them carried up to one antibiotic resistance gene, and the remaining 8 carried more than one antibiotic resistance gene. Survival was assessed using the plate count method. The tested pressure parameters from 300 to 600 MPa during 1, 3 or 5 min were efficient, and it was shown that the strains carrying antibiotic resistance genes showed a lower survival in response to high pressure values. Looking at the differences in the number of CFU/mL depending on the pressure used and the treatment time, statistically significant differences were only observed after applying a pressure of 400 MPa for 1 and 3 min. No significant differences in survival were observed between *Lactobacillus* and *Lactococcus* strains. It was concluded that this might be explained by the phenomenon of fitness cost, consisting of a reduced adaptation of antibiotic-resistant strains related to metabolic expenditure.

In the study by Nalepa et al. [4], the antimicrobial resistance was determined for twenty regional cheeses produced from non-pasteurized cow, goat and ewe milk. The phenotypic and genotypic antibiotic resistance of lactic acid bacteria isolated from these products was assessed. It was shown that among 79 LAB isolates, the most frequent species were *L. plantarum* (n = 18), *Leuc. lactis* (n = 17), *Lc. lactis* (n = 11), *Leuc. mesenteroides* (n = 9) and *L. pentosus* (n = 8). Additionally, *L. casei* was found in nine products. Analysis of phenotypic resistance to tetracycline (30 µg), erythromycin (15 µg), and chloramphenicol (30 µg) showed that 29% of LAB isolates were resistant to one antibiotic, 8% were resistant to two, and 12% were resistant to all tested antibiotics. Antibiotic resistance genes (AGR) for tetracycline (tet(M), tet(L), tet(W)), erythromycin (erm(B)) and chloramphenicol (cat-TC) were detected in 30 (38%), 29 (36.7%) and 33 (43.4%) LAB isolates, respectively. Among 31 LAB isolates phenotypically susceptible to all tested antibiotics, only 5 (16%) had no ARGs. It was concluded that a vast majority of tested LAB isolates had antibiotic resistance genes, and therefore the population of lactic acid bacteria found in regional cheeses can represent a potential source of ARGs in the environment. The issue of food microbiological safety is still a lively and important topic, and the more research in this area, the better. The use of tested and well-characterized starter cultures as well as the selection of raw material and culture is an important issue, especially from the point of view of developing novel, safe and nutritious food.

Also, the use of new additives in fermented products should be implemented in parallel with testing the adaptability of starter cultures. The use of supercritical extracts (scCO_2_/H_2_O) obtained from the mixture of bark and wood of black poplar (*Populus nigra*) and basket willow (*Salix viminalis*) as the source of bioactive compounds in yogurt production was tested by Walter et al. [6]. The addition of a supercritical *S. viminalis* extract reduced the time for natural and probiotic yogurt fermentation. Natural and probiotic yogurt with scCO_2_/H_2_O extracts added contained over 7 log CFU/g, and the LAB were active throughout the cold storage period. The incorporation of scCO_2_/H_2_O extracts at a dose of 0.01% (*w*/*v*) into milk for the production of natural and probiotic yogurts increases their functional properties by enhancing the antioxidant activity without causing negative effects on the physicochemical and organoleptic properties of products.

There is also one paper published in the Special Issue regarding the use of fungal flora for a direct rub inoculation of dry-aged beef (DAB) [5]. Mikami et al. [5] applied fungal flora for the preparation of portions of Holstein steers’ rumps. The crust was covered with fungal flora, including *Mucor flavus*, *Helicostylum pulchrum*, and *Penicillium* series *Camembertiorum*. Fungal-inoculated portions were compared to portions without treatment (conventional DAB) and dry-aged for 26 days in an ageing room at 2.9 °C and 90% relative humidity. The fungal covering and meat quality parameters, including fatty acid composition and volatile aromatic compounds, of fungal-inoculated DAB were compared with those of the conventional DAB. The fungal-inoculated DAB was almost entirely covered with white mold, in contrast to the conventional DAB. Moreover, the proportion of oleic acid and the concentration of nine volatile compounds significantly increased in fungal-inoculated DAB compared with the conventional DAB (*p* < 0.05). Notably, the level of 1-hexanol, which was previously described as having a “flower”, “green”, “nutty”, and “popcorn-like” flavor, was considerably increased. These results suggested that direct rub inoculation of fungal flora from prepared DAB may accelerate DAB production and efficiently enhance the “melt-in-the-mouth” feeling and flavors of DAB. Such targeted treatments with microbial components may improve the quality and safety of novel products.

The fatty acid composition was also tested by Paszczyk et al. [8,9] in goat milk-based and cow’s milk-based fermented products. The conducted research showed that cow’s milk-based natural yogurts with additives (muesli, cereal grains) had the highest content of polyunsaturated fatty acids (PUFAs) and n-3 PUFAs. Natural and bio yogurts with additives (millet groats, quinoa, chestnuts) had a higher content of n-6 PUFAs than the other analyzed yogurts. The n-6/n-3 ratio was lower in bio yogurts and eco yogurts. The fat extracted from the bio yogurts had the highest (0.90% of total fatty acids) mean content of cis9trans11 C18:2 (CLA). In fat of the other analyzed yogurts, the mean CLA content in the total content of fatty acids varied from 0.48% in natural yogurts with additives to 0.81% in bio yogurts with additives [8]. During the analysis of goat milk-based products, it was stated that raw organic goat’s milk had a significantly higher content of CLA (3.26 mg/g fat) compared to commercial goat milk (2.88 mg/g fat and 2.54 mg/g fat). Among the analyzed fermented goat’s milk drinks, the highest CLA content (4.39 mg/g fat) was determined in commercial natural yoghurts, while the lowest CLA content was observed in organic natural yoghurts (3.28 mg/g fat) [9]. The initial composition of the material and fermentation were found to play a significant role in the development of beneficial properties in fermented products.

Japanese-Saanen goats milk is also a source of probiotic strains that were thoroughly characterized by Tanaka et al. [10]. Among the 101 isolated positive candidates, two strains, YM2-1 and YM2-3, were selected and identified as *Lacticaseibacillus rhamnosus* using 16S rDNA sequence similarity. Culture supernatants of the two strains exhibited antipathogenic activity against *Salmonella enterica* subsp. *enterica serovar. Typhimurium, Shigella sonnei*, methicillin-resistant *Staphylococcus aureus*, methicillin-sensitive *Staphylococcus aureus*, *Listeria monocytogenes*, and *Escherichia coli* O_157_. Both YM2-1 and YM2-3 strains showed less unfavorable activities, including bile acid bioconversion, carcinogenic-related enzymes, mucin degradation, plasminogen activation, and hemolysis, than the detection limits of in vitro evaluation methods used in this study. In summary, *L. rhamnosus* YM2-1 and YM2-3 are highly safe and promising probiotic strains applicable in the dairy industry.

High-quality milk and meat are obtained from healthy animals, and therefore Zhao et al. [7] focused on the importance of the early feeding of calves. In this study, six healthy male Holstein calves received either colostrum or mature milk at 2 h and at 12 h after birth. In the collected samples of the mid-jejunum, phosphoproteome was analyzed using titanium-immobilized metal ion affinity chromatography, coupled with liquid chromatography-tandem mass spectrometry. It was shown that the phosphoproteins were strongly associated with developmental and macromolecule metabolic processes, signal transduction, and responses to stimuli and insulin. The results indicated the expression pattern and changes in the function of phosphoproteins in bovine jejunum tissues under different feeding conditions, as well as providing further insights into the role of colostrum feeding during the early stages of calves’ lives [7].

Finally, Merlino et al. [11] showed that consumers are increasingly aware of the benefits of local foods in terms of quality, sustainability, animal welfare, and safety. This research addresses two main questions: (1) is the perception regarding sustainability aspects of local dairy products related to individuals’ preferences for milk and cheese quality aspects? (2) Are these perceptions related to people’s socio-demographic characteristics? The availability request component described the consumer need for higher availability and visibility of local dairy products on the market. The locavorism in terms of fermented products is strongly declared. The effect of gender, age, and educational status of individuals emerged as significantly important for the resulting component definitions. The obtained results clearly suggest the need to increase the efficiency of communication strategies concerning local dairy products, as well as local dairy products’ availability and visibility on the markets.

Eleven articles published in this Special Issue presented recent developments and applications of fermentation and microbiota analysis to improve food quality and safety. Due to the inclusion of well-designed and well-written articles, this Special Issue offers a valuable contribution to the field of food technology. Although many relevant findings have been published in recent years, there is still a need for further improvements and developments of fermented products based on raw materials of animal origin. In particular, the issues related to the safety of such food require further intensive work.

## References

[1] Ogrodowczyk A.M., Jeż M., Wróblewska B. (2022). The Manifold Bioactivity and Immunoreactivity of Microbial Proteins of Cow and Human Mature Milk in Late Lactation. Animals.

[2] Elafify M., Sadoma N.M., Abd El Aal S.F.A., Bayoumi M.A., Ismail T.A. (2022). Occurrence and D-Tryptophan Application for Controlling the Growth of Multidrug-Resistant Non-O157 Shiga Toxin-Producing *Escherichia coli* in Dairy Products. Animals.

[3] Zarzecka U., Zadernowska A., Chajęcka-Wierzchowska W., Wiśniewska K., Modzelewska-Kapituła M. (2022). Antibiotic Resistance Carriage Causes a Lower Survivability Due to Stress Associated with High-Pressure Treatment among Strains from Starter Cultures. Animals.

[4] Nalepa B., Markiewicz L.H. (2023). Microbiological Biodiversity of Regional Cow, Goat and Ewe Milk Cheeses Produced in Poland and Antibiotic Resistance of Lactic Acid Bacteria Isolated from Them. Animals.

[5] Mikami N., Toyotome T., Takaya M., Tamura K. (2022). Direct Rub Inoculation of Fungal Flora Changes Fatty Acid Composition and Volatile Flavors in Dry-Aged Beef: A Preliminary Study. Animals.

[6] Walter M., Brzozowski B., Adamczak M. (2021). Effect of supercritical extract from black poplar and basket willow on the quality of natural and probiotic drinkable yogurt. Animals.

[7] Zhao X., Qi Y., Wu T., Cheng G. (2023). Phosphoproteomic Analysis of the Jejunum Tissue Response to Colostrum and Milk Feeding in Dairy Calves during the Passive Immunity Period. Animals.

[8] Paszczyk B., Czarnowska-Kujawska M. (2022). Fatty Acid Profile, Conjugated Linoleic Acid Content, and Lipid Quality Indices in Selected Yogurts Available on the Polish Market. Animals.

[9] Paszczyk B., Czarnowska-Kujawska M., Klepacka J., Tońska E. (2023). Health-Promoting Ingredients in Goat’s Milk and Fermented Goat’s Milk Drinks. Animals.

[10] Tanaka Y., Aryantini N.P.D., Yamasaki E., Saito M., Tsukigase Y., Nakatsuka H., Urashima T., Horiuchi R., Fukuda K. (2023). In Vitro Probiotic Characterization and Safety Assessment of Lactic Acid Bacteria Isolated from Raw Milk of Japanese-Saanen Goat (*Capra hircus*). Animals.

[11] Merlino V.M., Renna M., Nery J., Muresu A., Ricci A., Maggiolino A., Celano G., De Ruggieri B., Tarantola M. (2022). Are Local Dairy Products Better? Using Principal Component Analysis to Investigate Consumers’ Perception towards Quality, Sustainability, and Market Availability. Animals.

